# Finding Asymptomatic Spreaders in a COVID-19 Transmission Network by Graph Attention Networks

**DOI:** 10.3390/v14081659

**Published:** 2022-07-28

**Authors:** Zeyi Liu, Yang Ma, Qing Cheng, Zhong Liu

**Affiliations:** 1 College of Systems Engineering, National University of Defense Technology, Changsha 410073, China; lzylyn@hotmail.com (Z.L.); phillipliu@263.net (Z.L.); 2 Department of Mathematics and Statistics, University of Calgary, Calgary, AB T2N 1N4, Canada; 3 College of Systems Engineering, Aviation University of Air Force, Changchun 130000, China; yang_ma_cn@163.com

**Keywords:** COVID-19 transmission network, graph attention network, asymptomatic spreader, graph context loss function

## Abstract

In the COVID-19 epidemic the mildly symptomatic and asymptomatic infections generate a substantial portion of virus spread; these undetected individuals make it difficult to assess the effectiveness of preventive measures as most epidemic prevention strategies are based on the detected data. Effectively identifying the undetected infections in local transmission will be of great help in COVID-19 control. In this work, we propose an RNA virus transmission network representation model based on graph attention networks (RVTR); this model is constructed using the principle of natural language processing to learn the information of gene sequence and using a graph attention network to catch the topological character of COVID-19 transmission networks. Since SARS-CoV-2 will mutate when it spreads, our approach makes use of graph context loss function, which can reflect that the genetic sequence of infections with close spreading relation will be more similar than those with a long distance, to train our model. Our approach shows its ability to find asymptomatic spreaders both on simulated and real COVID-19 datasets and performs better when compared with other network representation and feature extraction methods.

## 1. Introduction

The COVID-19 epidemic has caused the most serious threat to global health since the early twentieth century. In this pandemic, health care authorities relied on preventive measures to reduce the spread of SARS-CoV-2 [1]. However, assessing the effectiveness of these preventive measures was difficult due to the presence of mildly symptomatic and asymptomatic individuals. These undetected individuals generated a substantial portion of disease spread due to SARS-CoV-2 viral shedding and transmission before the onset of symptoms [2,3]; thus, effectively identifying undetected patients in COVID-19 transmission networks will be of great help in disease prevention and control, especially in China, in which a dynamic zero-COVID-19 strategy was adopted. The cost of achieving this goal will be very high without the help of advanced technology to find these asymptomatic spreaders in local transmission when facing the Omicron mutant [4], although the rest of the world has mostly adopted a strategy of living with SARS-CoV-2 [5].

Recent advances in next-generation sequencing (NGS) platforms emphasize their application value in tracking emerging infectious disease outbreaks [6]. The combined approach of using genomic sequencing data with epidemiological data has successfully revealed transmission events for various viral outbreaks [7,8]. In determining critical features in the transmission pattern, such as the origin and the emergence of variants, viral sequencing can infer closely related isolates in an outbreak and identify unsampled cases in ongoing outbreaks [9]. Rapid viral sequencing can therefore provide real-time surveillance of transmission events and circulating viral variants in the ongoing COVID-19 pandemic. Network modeling tools such as Bayesian phylogenetics [10] and TransPhylo [9] have been utilized to capture the evolutionary and infection dynamics of SARS-CoV-2. Research using these tools have been able to establish phylogenetic pipelines using published SARS-CoV-2 genomic data to examine reasonable estimate transmission networks with the inference of unsampled infection sources. However, the computational cost is high for the calculation of Bayesian inference when dealing with large amounts of data.

Viral genomic approaches, including viral genomic sequencing and phylogenetic analyses, allow us to investigate fundamental characteristics in the transmission of an infectious disease. This is made possible by detecting the genetic variation in the viral genomes of infected individuals as a result of high rates of mutation and replication in transmission events [11]. Since RNA viruses have high mutation rates when they spread [12], the genetic sequence of the virus carried in each patient will be different; pertinently, SARS-CoV-2 is also one kind of RNA virus. As the mutation rate of SARS-CoV-2 is low [13], these species signatures of different subtypes are then passed on to those they infect, and all of the individuals in a local module in a network share common signatures. Our approach makes use of sequence variation within individuals. It is obvious that the genetic sequence of infections with close spreading relationships in a transmission network will be more similar than those with long distances. Based on this, the similarity of two neighbor nodes having asymptomatic or undiagnosed nodes between them must be lower than those without undetected nodes. Therefore, the main idea for finding asymptomatic spreaders in a COVID-19 transmission network is based on the similarity of each pair of neighbor nodes in the network.

By using a long short-term memory (LSTM) network, which is a deep neural network for modeling sequential data [14,15], the proposed model can learn the sequential information contained in these subgraphs for each target node. This information will be combined into a new embedding by an attention mechanism [16,17], and the embedding also captures information of the graph structure. As we expect nearby nodes to have similar representations and distant nodes to have dissimilar representations, graph context loss function, which is well matched with the characteristics, is used to train this model. By using our trained model to measure the similarity—the distance in the embedding space—between pairs of nodes via their representations, we can discover which pairs are unusually different for their given location in the transmission network, indicating that there are undetected nodes in between.

We first test our model on simulated datasets. The simulation transmission network is generated based on the rule of virus spread and the corresponding genetic sequence is simulated according to the characteristics of the SARS-COV-2 gene and the mutation when it spreads. The transmission network and gene sequence datasets are used to train our model. Then we randomly remove a certain proportion nodes and reconnect it to form a test network, the new connected edges, being removed nodes, form the test label set. Through different kinds of experiments, RVTR can effectively find undetected nodes in simulation transmission networks. We further show the model’s performance in real situations by training and testing the model on a COVID-19 dataset from Australia. The prediction of our proposed model is better than other comparison algorithms. Of note, more experiments have been performed on datasets from Canada, Alberta, New York State and New Zealand; all these experimental results indicated the model’s ability to find asymptomatic spreaders in SARS-CoV-2 transmission networks.

## 2. Methods

### 2.1. Background

The goal of finding asymptomatic spreaders is to infer undetected nodes in a COVID-19 transmission network, and our approach is based on the representation of the transmission network. In this paper, the goal of network representation is to correctly express the gene sequence information and network transmission characteristics of nodes in low dimensions, and then use the new representation information to discover undetected nodes.

#### 2.1.1. COVID-19 Transmission Network Representation

In a COVID-19 transmission network, virus transmitters are regarded as the network nodes, and the genetic sequence of SARS-CoV-2 can be regarded as the attribute of each node. A COVID-19 transmission network can be expressed as G=(N,E,A), where *N* is a set of nodes in the network. *E* represents the set of connected edges, and each edge $(n_{i},n_{j})\in E$ means that the virus is transmitted from node $n_{i}$ to node $n_{j}$. *A* represents the node attribute set, $a_{i}\in A$ represents the gene sequence of node $n_{i}$.

#### 2.1.2. Undetected Nodes and Abnormal Edges

**Figure 1:**
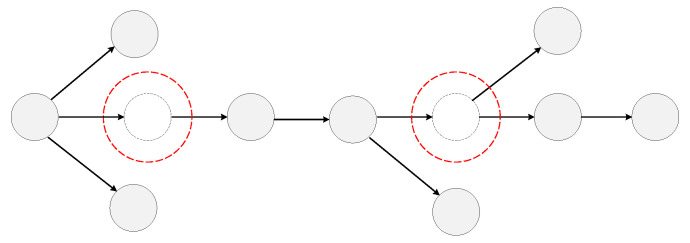
A complete COVID-19 transmission network. The dotted nodes marked with red circles are unsampled or asymptomatic spreaders.

Due to these asymptomatic or unsampled patients, the amount of detected COVID-19 infections is smaller than the actual number in the transmission network. Figure 1 shows a complete transmission network, but mostly it could not detect unsampled or asymptomatic spreaders, which are marked by red dotted circles. In this paper, we name these asymptomatic or unsampled infections undetected nodes for convenience.

**Figure 2:**
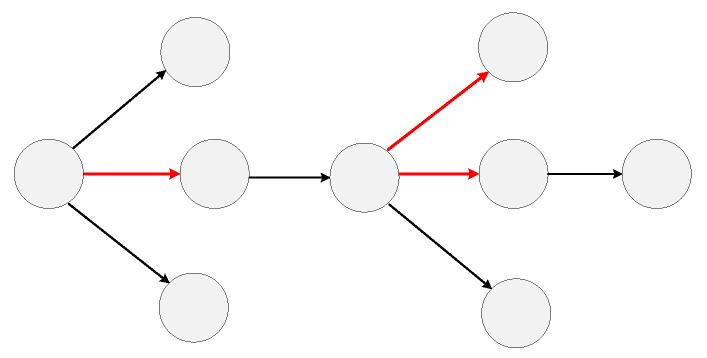
The observed COVID-19 transmission network. The red edges are abnormal transmission relations with undetected nodes in it.

These undetected infections lead to abnormal connections in an observed transmission network. As shown in Figure 2, the parent nodes and child nodes that should not have a direct relationship but produce the connection are marked by red edges. Finding asymptomatic or unsampled infections could be seen by finding abnormal edges in a transmission network. In our approach the representation of an RNA virus transmission network is learned first and then node similarity, which has a connection, is calculated. Finally, we use these similarity scores to find abnormal edges and locate the undetected infections in a transmission network.

### 2.2. Model

**Figure 3:**
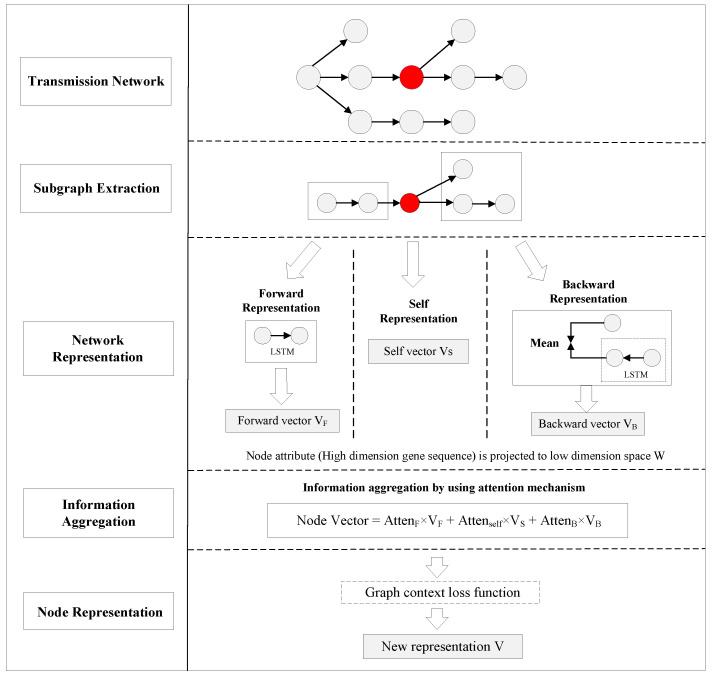
The framework of RVTR.

In this section, we introduce the RNA virus transmission network representation model based on the graph attention network (RVTR). The framework of the RVTR is depicted in Figure 3, in which a red node serves as an example of how this model generates a new representation by learning the information of neighbor nodes.

#### 2.2.1. Subgraph Extraction

Subgraph extraction aims to create a node set with the highest correlation for the target node, to ensure that the model can extract enough topology information from the transmission network. Previous studies [18,19] showed that the influence between nodes with a distance of more than three steps in a network is small, and we select nodes less than three steps from the target node $n_{i}$ to form subgraphs for the target node in the RVTR with computing efficiency. As shown in Figure 3, nodes, except the target node, are divided into two categories—forward set $FS_{n}$ and backward set $BS_{n}$, based on the propagation relationship. The forward set $FS_{n}$ contains the second-order afferent nodes of the target node, namely the parent node and the grandfather node of the target node. The backward set $BS_{n}$ is the second-order efferent nodes of the target node, namely the child nodes and the grandchild nodes of the target node.

#### 2.2.2. Subgraph Representation

As shown in Figure 3, subgraph representation aims to learn a lower dimension vector for the incoming node set and outgoing node set. In the calculation of forward representation, due to the propagation relationship between nodes, we used LSTM to aggregate forward information, and selected the last hidden layer of LSTM as the forward vector $V_{F}$. When calculating the backward representation, we adopted the backward LSTM method similar to the forward representation for each child branch. The last hidden layer of LSTM was selected as the representation vector for each branch, and we took the mean value of all branch vectors as the backward representation vector $V_{B}$. For the target node itself, the self-representation $V_{S}$ was converted to the specific dimension through a multilayer full connection layer.

(1) $$\begin{array}{c} V_{F}\left(n\right)=\vec{LSTM_{F}}\left(BN\left(FC\left(e_{F}\right)\right)\right),n\in FS_{n}\\ V_{B}\left(n\right)=\overset{\leftarrow }{LSTM_{F}}\left(BN\left(FC\left(e_{B}\right)\right)\right)/\left|S\left(n\right)\right|,n\in FS_{n}\\ V_{S}\left(n\right)=BN\left(FC\left(e_{S}\right)\right). \end{array}$$

Among these variables, BN represents the batch normalization operation, FC represents the full connected layer, and these three vectors are denoted as *V*, which have the same dimension. Through this operation, the model obtains the representation of the structural information extracted from the network subgraph for each node.

#### 2.2.3. Information Aggregation Based on Graph Attention Mechanism

Information aggregation aggregates the representation of each part in the subgraph. We used the self-attention mechanism [16] to learn the aggregation weight of the forward, self and backward representation vectors $V_{k}$, and k∈{F,S,B}; then, we can obtain an aggregated vector $V_{n}$ for each node.

(2) $$\begin{array}{c} V=AGGREGATION(V_{F},V_{S},V_{B})\\ =\alpha_{F}\cdot V_{F}+\alpha_{S}\cdot V_{S}+\alpha_{B}\cdot V_{B}. \end{array}$$

The corresponding attention values $\alpha_{k},k\in F,S,B$ and $\alpha_{k}$ are learnable parameters as follows:

(3) $$\begin{array}{c} \alpha_{k}=\frac{exp(\sigma \left[a^{T}\cdot \left(V_{S}⨁V_{k}\right)\right])}{\sum_{l\in F,S,B}exp\left(\sigma \left[\alpha_{l}^{T}\cdot \left(V_{S}⨁V_{l}\right)\right]\right)}, \end{array}$$

where ⨁ is the concatenation operation and $a^{T}\in R^{2d\times 1}$ and $a_{l}^{T}\in R^{2d\times 1}$ are the learnable attention parameters. σ is the LeakyReLU function.

#### 2.2.4. Graph Context Loss Function

Considering the mutation of SARS-CoV-2 when it spreads, it is obvious that the similarity of neighbor nodes’ gene sequences is higher than that of non-neighbor nodes. To achieve this goal, the graph context loss function [20] is well matched with the characteristics of the virus transmission network. The loss function transformation is defined as:

(4) $$\begin{array}{c} loss=-\sum_{<n,i,j>}\left[log\theta \left(V_{n}\cdot V_{i}\right)+log\theta \left(-V_{n}\cdot V_{j}\right)\right]+{\beta ||w||}_{2}^{2}, \end{array}$$

where $V_{n}$ is the output node embedding formulated by the RVTR. Among them, 〈n,i,j〉 is a triple, n∈N is the target node, $i\in W_{n}$ is the context neighbor in graph *G*, j∈N are the negative sampling nodes, ${\left||w|\right|}_{2}^{2}$ is the L2 regularization function, β is the weight parameter. Specifically, we used a random walk to obtain the set of context nodes for each node, with a restart probability of $p_{r}$, and a walk length is $L_{w}$. Then a negative node $j(j\notin W_{n})$ was sampled randomly from the network. To improve the computational efficiency, we chose a specific proportion of nodes for sampling during each generation of training.

#### 2.2.5. Similarity Calculation

After obtaining the new representation *V* for each node, we can evaluate whether the edge is abnormal by calculating the similarity of its two corresponding nodes. For nodes *i* and *j*, the similarity is calculated as follows:

(5) $$\begin{array}{c} S_{ij}=V_{i}\cdot V_{j}. \end{array}$$

After obtaining the similarity scores for all the edges in the transmission network, these edges whose scores are relatively low are more likely to be abnormal.

### 2.3. Experimental Design

#### 2.3.1. Two Kinds of Test Experiments

To show the performance of the RVTR, we performed two kinds of test experiments. One was training and testing on the same network, and the other was training and testing on different networks. In the simulation experiment, first, we generated and trained our model in this network. Then, we randomly removed certain nodes from it and rebuilt the network. The new reconstructed network was our test work. In the other experiment, some different networks were generated for testing; the steps to generate the test label were the same as those in the first one. Similar to the simulation experiment, we first trained and tested on the Australia dataset in the real data experiment, and then we tested the RVTR on different datasets.

#### 2.3.2. Comparison Algorithms

The RVTR is based on a virus transmission network that encodes both the graph structure and features of nodes. It is one kind of network representation method; therefore, two network representation methods, graph convolutional neural network (GCN) [21] and structural deep network embedding (SDNE) [22], were selected for comparison. In this work, we used a two-layer GCN model, in which the dimension of the hidden layer and the dimension of the output layer were the same as those of the RVTR. We used a three-layer neural network in SDNE, the dimensions of two hidden layers are 1024 and 512, and the dimension of the output layer is the same as that of the RVTR. Besides, RVTR also reduces the dimension of the attributes of the network, so we also chose to perform principal component analysis (PCA) [23] and autoencoder (AE) [24], which can reduce the dimension and extract features for high-dimensional gene sequences. In PCA, the principal component of the gene sequence was used as the input of the task of finding undetected nodes. The output dimension of PCA was the same as that of the RVTR model.  AE was used to learn a representation for a gene sequence directly without considering the network structure. We first trained the AE model in a manner similar to that used for our model, and then the middle layer of the trained AE model, namely, the sequence representation after dimensionality reduction, was used as the input of the task of finding missing nodes. In addition, we used a ‘DIRECT’ method, which means that the high-dimensional gene sequence was used for calculation directly without any loss. The description of the comparison algorithms is shown in Appendix A.1 of Appendix A, the setting of RVTR is described in Appendix A.2 of Appendix A.

#### 2.3.3. Evaluation Metrics

It should be noted that, as the RVTR is an unsupervised learning method, it cannot directly predict the number of abnormal edges. To calculate the prediction accuracy, we set the model to predict the same number of abnormal edges as the label set, and then we compared the prediction results with true labels. Precision is calculated by comparing the prediction with the true label and the proportion of correct predictions in all labels.

(6) $$\begin{array}{c} Precision=\frac{m}{k}, \end{array}$$

where *m* is the amount of correct prediction, and *k* is the number of abnormal edges in the label set.

As RVTR is a kind of network representation method, we also calculated the AUC value [25], which is commonly used to measure the effect of algorithms in link prediction, to evaluate the performance of different models.

## 3. Materials

### 3.1. Simulation Data

First, we needed to design a simulation experiment to evaluate the performance of the RVTR model as it was difficult for us to obtain a complete COVID-19 transmission network to train our model in real situations. The simulated dataset was generated based on the character of the SARS-CoV-2 virus gene and spread to test our model first.

#### 3.1.1. Training Data Generation

- Sequence simulation

We set the length of the simulated SARS-CoV-2 gene to *L*. According to the in-house filter present in GISAID, complete sequences were comprised of genomes with lengths greater than 29,000 nucleotides [26]; here, we set *L* as 30,000. For the value of each gene, we used A,T,G and *C* to generate the whole gene sequence at each position randomly. Although there were missing symbols ‘ ’ or gap symbols ‘-’ in a real sampled sequence in a real situation as a limitation of high-throughput genome sequencing, we did not consider this situation for simplicity in simulation experiments. Assuming that each variation per gene is independent, the mutation rate of the whole sequence at each transmission was *p*. Regarding the mutation rate of SARS-CV-2 being low when it spreads [13], here, we set p=0.1%.

- Transmission network simulation

**Figure 4:**
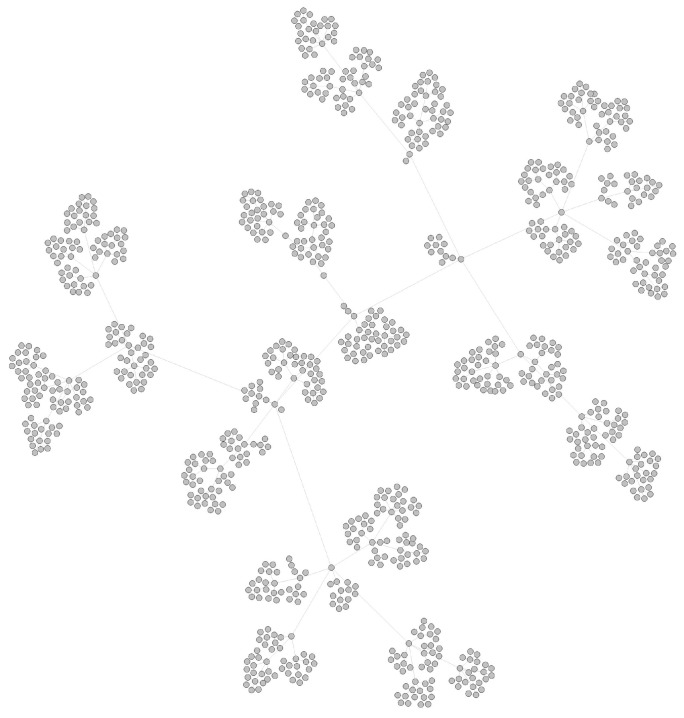
A simulated COVID-19 transmission network with 1000 nodes.

As the value of the basic reproduction number (denoted R0) of SARS-COV-2 is approximately three in the early stages, here we set the range of *R* from 0 to 6, in which *R* represents the number of child nodes created when we simulate the transmission network in one generation. The value of *R* at each transmission belongs to a Poisson distribution. To simplify, we set a fixed value, the probability $p_{\left(R=0\right)},p_{\left(R=6\right)}=5\%$, $p_{\left(R=1\right)},p_{\left(R=5\right)}=10\%$, $p_{\left(R=2\right)},p_{\left(R=4\right)}=20\%$, $p_{\left(R=3\right)}=30\%$. Assuming that all nodes in the network start from “patient 0”, the pseudocode of generating a simulated COVID-19 transmission network and its corresponding gene sequence is described in Algorithm 1. A simulated transmission network that has 1000 nodes is shown in Figure 4. We generated three training networks with 1000, 2000 and 3000 nodes.
**Algorithm 1** Generating procedure of simulated data.**Input:** Model parameters *N*, $R_{n}$, *L*, *p***Output:** Transmission network and the corresponding gene sequence**while**i<L**do**   randomly choose A, T, G or C**end while****Return** gene sequence for node $n_{0}$**while**j<N**do**   generate child nodes by choosing an R0 value from the range of R0 values following the probability for each value   **while** $k<R_{n}$ **do**     copy the gene sequence of node $n_{0}$     Randomly change the p×L gene to generate a gene sequence for child nodes   **end while**   **Return** gene sequence of each node**end while****Return** transmission network

#### 3.1.2. Test Data Generation

**Figure 5:**
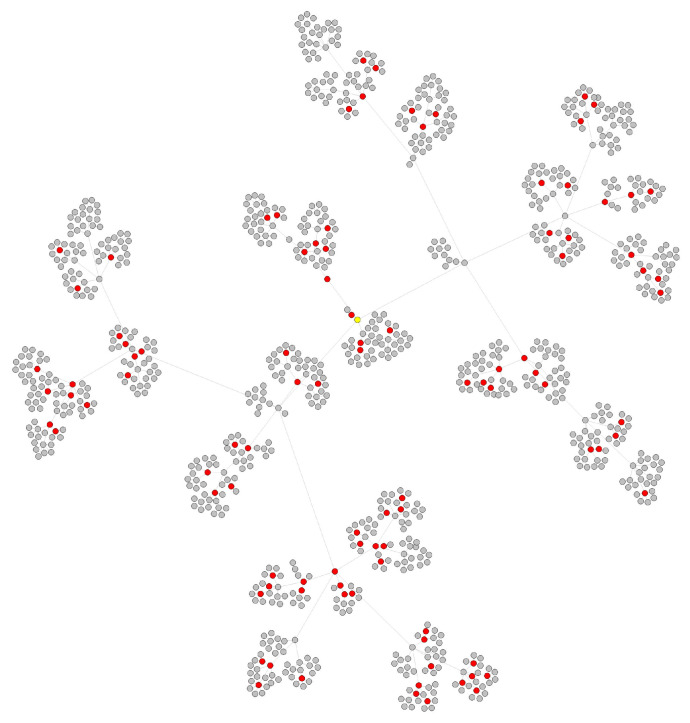
Ten percent of the nodes are selected as undetected nodes that are marked by red. They will be removed from the network.

**Figure 6:**
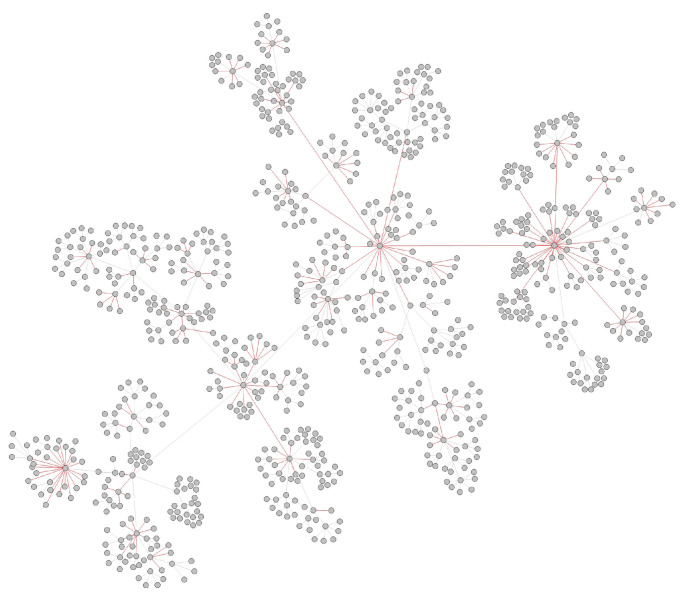
Reconnected network, red edges are abnormal edges with undetected nodes in them.

To show the performance of the RVTR model, we simulated a test network similar to the transmission network detected in a real situation. We randomly chose and removed some nodes from a transmission network, and these removed nodes were considered undetected nodes. Then, the network was rebuilt, and the reconnected edges were abnormal edges and marked as our true labels to calculate the prediction accuracy. As nodes at the margin of the network indicate the end of virus spread, it did not make sense to choose these nodes as undetected nodes. The pseudocode of test data generation is shown in Algorithm 2. The red nodes in Figure 5 are the nodes selected to be removed from the network in Figure 4. We removed 10% of the nodes from the network, and the number of red nodes is 100. Figure 6 shows the reconnected network after removing the red nodes. The red edges are abnormal edges, and they form a label set to test the accuracy of RVTR’s predictions.
**Algorithm 2** Test transmission network and label data generation.**Input:** a transmission network, the proportion of removed nodes $p_{r}$**Output:** a test network, label data set**while**$i<p_{r}\times N$**do**   randomly choose $p_{r}\times N$ nodes in the network**end while****if** the selected node is at the margin of the network **then**   retain it in the network**else**   remove it from the network   connect the parent node of the removed node to its child nodes   Add new connected edges into the label set**end if****Return** a test network and label data set

### 3.2. Real Data

#### 3.2.1. Data Resource

As it is difficult to obtain a complete transmission network with undetected infections in real situations, we used the transmission networks inferred by Perera’s work [27], which used published SARS-CoV-2 genomic data to estimate reasonable transmission networks with the inference of unsampled infection sources. For the gene sequence data, we used the FASTA data, which was also used to infer the transmission network. The selected FASTA sequence data of Canada, New Zealand, New York State and Australia were downloaded from GISAID (https://www.gisaid.org/ Accessed on 1 September 2020) [26]. The Alberta dataset was obtained from the Provincial Laboratory of Alberta. The details of these datasets can be seen in Appendix A.3 of Appendix A.

#### 3.2.2. Real Data Processing

There are sequences with labels that contain complete collection dates and locations in the FASTA data. Before putting the FASTA sequence data into our model, we needed to transfer the labels corresponding to the inferred transmission network. In addition, the symbols ‘,’ between each gene also needed to be cleaned before being put into the models. Moreover, the assumption is that all infections have a common ancestor [28] that uses the Bayesian program TransPhylo, which is a dedicated software designed to reconstruct transmission networks from timed phylogenetic data to infer transmission trees. Therefore, the inferred spread started from patient “0”. However, it is almost impossible to find patient “0” in an area during the pandemic, so we needed to create a gene sequence for it. Moreover, as the length of the sequence in the FASTA data is slightly different for different datasets, we needed to adjust the sequence of different datasets for alignment. The details of the real data process and figures of these transmission networks can be seen in Appendix A.4 of Appendix A.

**Figure 7:**
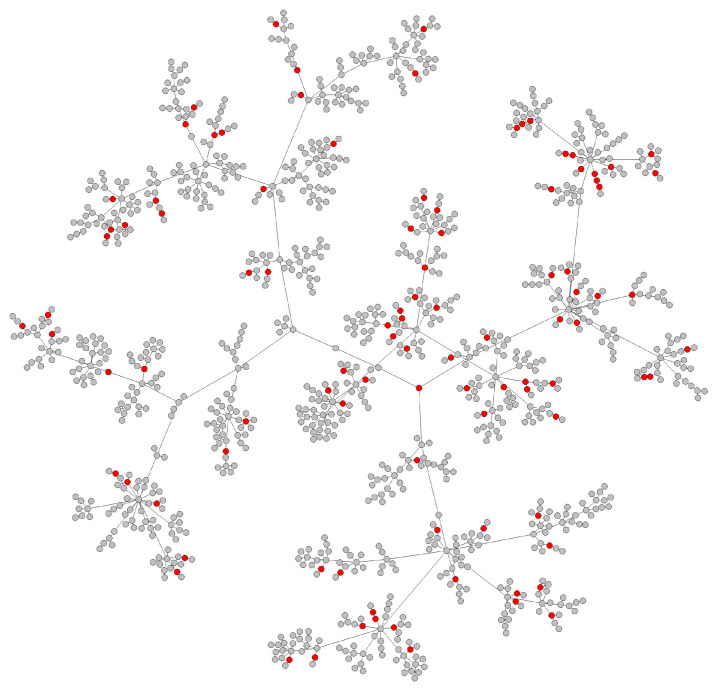
The inferred Australia transmission network, the red nodes are inferred undetected infections in Australia.

The spread of SARS-CoV-2 varies from region to region due to the different control measures in the COVID-19 pandemic, and the distribution of transmission data, such as the reproductive number, differs. We initially evaluated the inferred transmission data of Canada, Australia, Alberta, New Zealand, and New York State for testing the methodology. The details of these datasets are shown in Table 1. As the undetected infections of the Australia dataset are relatively small in these datasets, we chose the Australian dataset, for which the transmission network is relatively complete, as our training dataset. Figure 7 shows the inferred transmission network of Australia.

**Figure 8:**
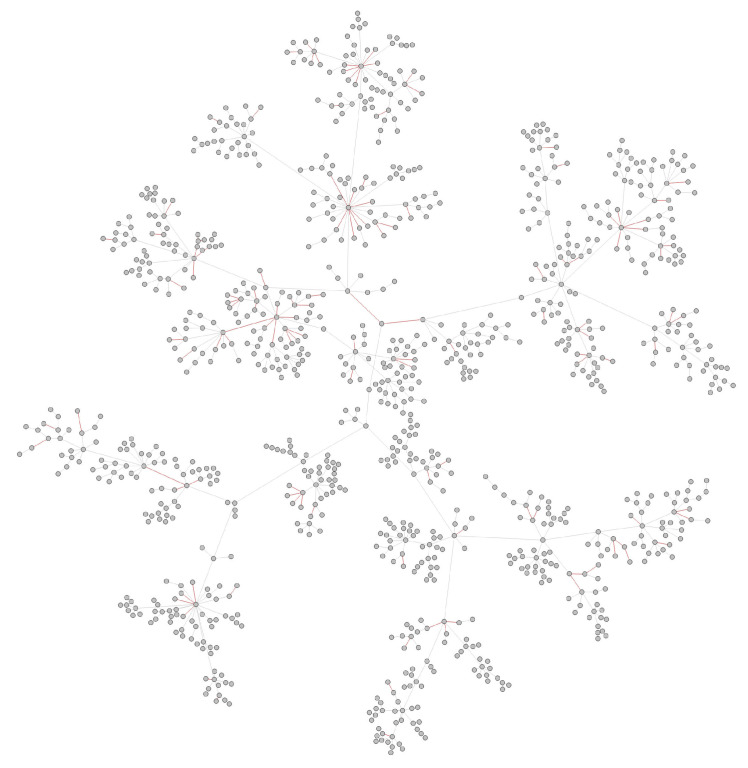
Abnormal edges in Australia transmission network.

Considering the inferred transmission networks contain undetected nodes, we needed to remove the inferred unsampled infections before using them to test. Similar to the process in the simulation experiment, the reconstructed network was our test network, and the new reconnected abnormal edges constituted our label set. Figure 8 shows the inferred transmission network of Australia.

## 4. Results

### 4.1. Simulation Experiment Results

We compared the performance of the RVTR model with that of other methods on networks in which the initial sizes were 1000, 2000 and 3000. Test networks were generated by removing specific proportion nodes from initial networks. As the removed nodes were chosen randomly and the test network was different in each generation of the test network, we performed test experiments 10 times to prevent an uneven distribution of test data. The prediction results are shown as the mean value and variance of 10 results. As the RVTR, GCN and SDNE are all network representation methods, to be fair, we trained and tested the models on the same network in this experiment. The test results on networks of 1000, 2000 and 3000 are shown in Table 2, Table 3 and Table 4, respectively. The best results are highlighted in bold

After we compared the performance of the RVTR model with that of other methods through training and testing them on same networks, we tested the RVTR model by using different datasets to test its ability. We trained two different RVTR models: RVTR-1K, which was trained on a network with a size of 1000, and RVTR-3K, which was trained on a network with a size of 3000. Then, the trained model was used to test on networks of different sizes and the results are shown in Table 5. The best results are highlighted in bold.

### 4.2. Real Experiment Results

Although the transmission network with inferred undetected infections implemented in TransPhylo had been proven reasonable in previous work on an HIV dataset [28], it is hard to prove the inference is absolutely correct. To show the RVTR’s ability to find abnormal edges and capture the location of undetected nodes, we first trained and tested our model on the same network, and we randomly removed some nodes to perform tests similar to the simulation test experiments. Regarding the parameter settings for the real dataset experiment, we set the dimension of the output as 256, and the other parameters of the RVTR were the same as the parameters used in the simulation experiment. The best trained RVTR model is achieved among 2400 epochs and 3000 epochs.

Table 6 shows the test results on 10%, 20%, and 30% of the nodes removed from the Australia network, the best results are highlighted in bold. The results show that RVTR performed the best in finding undetected nodes in a real COVID-19 transmission network. The DIRECT method, which performed the best in the simulation experiment, cannot achieve a good performance in the real data experiment; this outcome may be related to the large invalid gene in the sequence, and it is necessary to extract the key features from the high-dimensional gene sequence.

After we trained and tested on the Australia dataset to prove the ability of the RVTR model to find undetected spreaders in a real transmission network, we tested the proposed model on four datasets of different regions. The prediction results can be seen in Table 7, the best results are highlighted in bold. From the test results on different datasets, we can see that the RVTR model achieves the best performance on transmission networks for almost all regions except New Zealand. According to the analysis of the New Zealand dataset, described in Appendix A.3 of Appendix A, the sampled infections in New Zealand are quite different from those in other datasets. SARS-CoV-2 barely spread in May, June and July 2020, although the duration of spread lasted more than one year, and the inferred transmission determined by TransPhylo may be questionable when compared with other datasets. The results also show that the prediction is better when there are more undetected nodes in the transmission network, such as in the New York and New Zealand datasets.

## 5. Discussion

From the precision value of prediction results in Table 2, Table 3 and Table 4, we can see that RVTR performs better than other network representation methods and feature extraction methods, although the best result was achieved by the DIRECT method. This may be related to the mechanism of sequence generation; embedding in lower dimensions will reduce the features of the simulation sequence. From the AUC value in Table 2, we can see that the AUC values of these models are also good when the precision values are great. However, good AUC values cannot guarantee fine precision values from the results of the RVTR on different test networks.

We can see that the model trained on a large network has a better performance when tested on different networks from the results in Table 5. From these simulation experiments, we can see that the performance of all algorithms is better when the removed proportion is larger, which means that the difference in sequence is larger when the transmission distance is longer. The more undetected nodes existed between two nodes, the lower the similarity score of this pair of nodes and it will be much easier for the RVTR to detect the difference. To show the performance of the RVTR in more detail, we also analyze the training details of RVTR and the influence of different network structures in this section.

### 5.1. Training Details of Simulation Experiment

**Figure 9:**
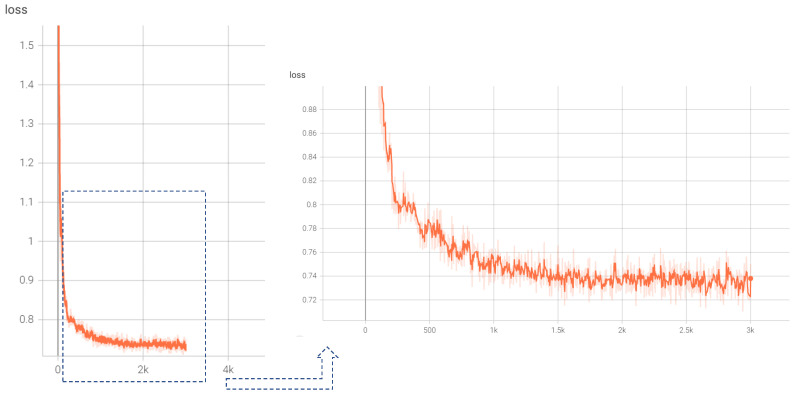
The change in the loss value in 3000 steps of model training.

Figure 9 shows the detailed loss change in the RVTR in the training step. We can see that the loss value drops rapidly at the early training stage, and then the change in the loss value becomes stable after 1000 epochs.

**Figure 10:**
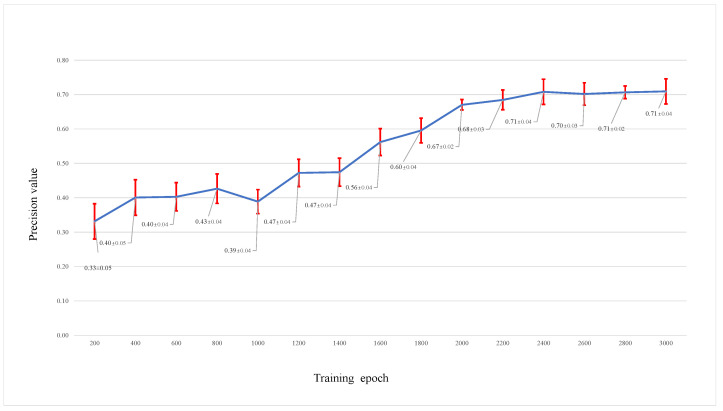
The prediction accuracy by trained models saved at different training epochs, the blue line shows the trend of predicion accuracy by trained models saved at different training epochs, the red line shows the range of standard err.

**Figure 11:**
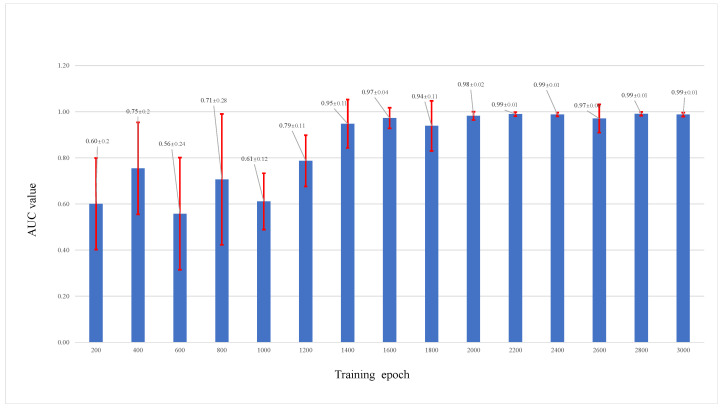
Different AUC values of trained models saved at different training epochs, the red line shows the range of standard err.

We also saved the trained parameters of the models every 200 epochs when training; then, these different trained models were used to test and change the performance of the different models. Figure 10 shows the test results of different trained models on test networks with an initial size of 1000 and 10% nodes removed. We can see that the prediction accuracy increases when the training epoch increases. The changes in the AUC value in Figure 11 also show a good performance when the RVTR is well trained.

### 5.2. The Influence of Network Structure

Based on [29], the asymptomatic nodes are connected differently from connections of other symptomatic nodes. For example, asymptomatic infections usually cause super spread as they do not show any symptoms and will contact with others as usual, while symptomatic infections will quarantine themselves and reduce the spread. The sensitivity of the results may be related to the selected nodes for removal and also the network structure, we also simulated two different transmission networks based on different *R* values to analyze the influence of network structure. The R value of the first one is approximately equal to 1.7, which is generated based on a fixed probability $p_{\left(R=1\right)}=50\%$, $p_{\left(R=0\right)},p_{\left(R=2\right)}=15\%$, $p_{\left(R=3\right)},p_{\left(R=4\right)},p_{\left(R=5\right)},p_{\left(R=6\right)}=5\%$; we named this network N1.7. The R value of the second one is approximately equal to 4.8, which is generated based on a fixed probability $p_{\left(R=6\right)}=40\%$, $p_{\left(R=5\right)}=30\%$, $p_{\left(R=4\right)}=15\%$, $p_{\left(R=1\right)},p_{\left(R=2\right)},p_{\left(R=3\right)}=5\%$, $p_{\left(R=0\right)}=0\%$, we named this network N4.8. To be fair, the RVTR is trained on a network of size 3000 and then tested separately on different networks. From the results shown in Table 8, we can see the RVTR achieves different performance on test networks with different transmission structures, the best results are highlighted in bold. The results are bad when the model is tested on the network with lower *R* value, while the predictions are good on networks with a higher *R* value.

**Figure 12:**
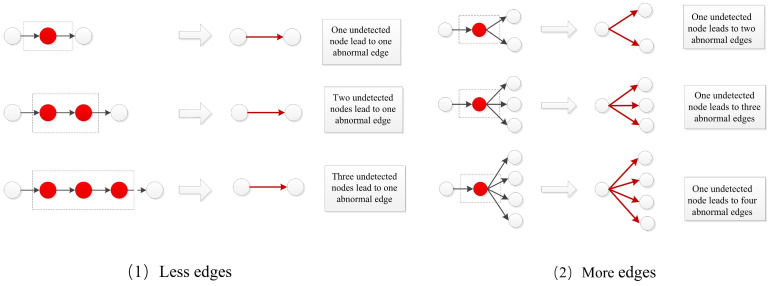
Two kinds of abnormal edges in real transmission network.

The reason for the prediction accuracy in Table 7 fluctuating greatly in real dataset experiments is related to the distribution of the abnormal edges. Figure 12 shows that different transmissions result in different kinds of abnormal edges. One outcome is “fewer edges”, which means that there is more than one undetected node in one abnormal edge. The outcome of “more edges” means that there is a “superspreader” in the detected transmission network. Although there were many abnormal edges in the sampled transmission network, there was only one undetected node. From Table 1, we can see that the distributions of the data for New Zealand and New York was similar; they both were of the “fewer edges” category. The distribution of the data for Alberta and Australia belonged to the “more edges” category, which means that there was a “superspreader” in the transmission network. In the first kind of abnormal edge, the difference in similarity of the two nodes was large, and the precision of finding the location of undetected nodes in the network is high. In the second kind of abnormal edge, the similarity value of these abnormal edges was close to the normal edge, as only one undetected node exists in them.

## 6. Conclusions

In this work, we propose a graph attention-based RNA virus transmission network representation model, i.e., RVTR, to find asymptomatic spreaders in the COVID-19 transmission network. The RVTR model achieves a good performance not only in simulated datasets but also in real COVID-19 transmission networks that were inferred by TransPhylo; we can see the ability of RVTR to find the location of undetected nodes in COVID-19 transmission networks. It means that we can use the RVTR model to find where undetected infections exist in this network after we construct a real COVID-19 transmission network by detected infections. For some areas and countries such as China, Singapore and Japan that take strict epidemic prevention and control measures, the epidemic departments usually conduct tracing extensively and publish detailed records of more than thousands of anonymized patients. A huge cost will be taken on to find the asymptomatic or undetected spreader hidden in transmission networks when some new infections are detected in an area. Our proposed method can be used to reduce the cost if it can tell where undetected infections exist in a transmission network, workers of epidemic control can focus on looking for asymptomatic spreaders in specific transmission relationships instead of looking for undetected spreaders across the entire network. Not only can it reduce the cost of conducting nucleic acid testing for all people in a region, but it can also save time to find the undetected spreader more quickly, thereby controlling the spread of COVID-19. However, as the RVTR is trained based on graph context loss, which entails unsupervised learning, it has the ability to find the locations of undetected nodes in the network but cannot tell us how many of them are present. In future work, we will change the loss function to use supervised learning by giving each edge a label with the number of undetected nodes in it; then, the proposed model will provide more information to help control the spread of SARS-COV-2.

## Figures and Tables

**Table 1:** The number of sampled nodes, inferred undetected nodes, and inferred abnormal edges in real datasets. Australia is represented as AU, New Zealand is represented as NZ, Canada is represented as CA, Alberta is represented as AB, New York State is represented as NY.

Dataset	AU	NZ	CA	AB	NY
Nodes	1031	618	964	1847	1581
Undetected nodes	100	845	539	733	3031
Abnormal edges	113	439	526	828	1412

**Table 2:** The prediction results by different methods tested on the network of 1000. The tests were performed on 10%, 20% and 30% nodes removed, respectively.

Removed	Metric	RVTR	GCN	SDNE	AE	PCA	DIRECT
10%	Precision	**0.71 ± 0.04**	0.31 ± 0.02	0.33 ± 0.04	0.33 ± 0.04	0.62 ± 0.02	**0.99 ± 0.00**
	AUC	0.98 ± 0.01	0.53 ± 0.48	0.50 ± 0.00	0.50 ± 0.00	0.82 ± 0.23	0.98 ± 0.01
20%	Precision	**0.91 ± 0.01**	0.59 ± 0.05	0.59 ± 0.04	0.60 ± 0.03	0.88 ± 0.01	**0.99 ± 0.00**
	AUC	0.97 ± 0.04	0.61 ± 0.41	0.50 ± 0.00	0.50 ± 0.00	0.87 ± 0.17	0.98 ± 0.00
30%	Precision	**0.98 ± 0.01**	0.89 ± 0.01	0.89 ± 0.01	0.89 ± 0.01	**0.98 ± 0.01**	**0.99 ± 0.00**
	AUC	0.97 ± 0.04	0.57 ± 0.23	0.50 ± 0.00	0.50 ± 0.00	0.93 ± 0.05	0.98 ± 0.01

**Table 3:** The prediction results by different methods tested on the network of 2000. The tests were performed on 10%, 20% and 30% nodes removed, respectively.

Removed	Metric	RVTR	GCN	SDNE	AE	PCA	DIRECT
10%	Precision	**0.68 ± 0.03**	0.30 ± 0.02	0.30 ± 0.04	0.30 ± 0.03	0.60 ± 0.02	**0.99 ± 0.00**
	AUC	0.97 ± 0.04	0.52 ± 0.32	0.50 ± 0.00	0.50 ± 0.00	0.53 ± 0.24	0.97 ± 0.01
20%	Precision	**0.85 ± 0.02**	0.60 ± 0.02	0.62 ± 0.03	0.63 ± 0.03	0.87 ± 0.01	**0.99 ± 0.00**
	AUC	0.95 ± 0.05	0.68 ± 0.44	0.55 ± 0.14	0.50 ± 0.00	0.63 ± 0.18	0.98 ± 0.00
30%	Precision	**0.98 ± 0.01**	0.91 ± 0.01	0.92 ± 0.01	0.92 ± 0.01	0.98 ± 0.01	**0.99 ± 0.00**
	AUC	0.98 ± 0.02	0.45 ± 0.40	0.70 ± 0.24	0.50 ± 0.00	0.80 ± 0.11	0.98 ± 0.01

**Table 4:** The prediction results by different methods tested on the network of 3000. The tests were performed on 10%, 20% and 30% nodes removed, respectively.

Removed	Metric	RVTR	GCN	SDNE	AE	PCA	DIRECT
10%	Precision	**0.69 ± 0.01**	0.33 ± 0.03	0.34 ± 0.01	0.33 ± 0.02	0.58 ± 0.03	**0.99 ± 0.00**
	AUC	0.96 ± 0.09	0.50 ± 0.41	0.50 ± 0.00	0.50 ± 0.00	0.76 ± 0.18	0.96 ± 0.05
20%	Precision	**0.89 ± 0.01**	0.63 ± 0.02	0.61 ± 0.03	0.60 ± 0.02	0.84 ± 0.01	**0.99 ± 0.00**
	AUC	0.98 ± 0.01	0.75 ± 0.38	0.49 ± 0.01	0.50 ± 0.00	0.76 ± 0.08	0.98 ± 0.01
30%	Precision	**0.98 ± 0.01**	0.92 ± 0.01	0.93 ± 0.01	0.93 ± 0.01	0.98 ± 0.01	**0.99 ± 0.00**
	AUC	0.99 ± 0.01	0.31 ± 0.05	0.50 ± 0.00	0.50 ± 0.00	0.83 ± 0.08	0.98 ± 0.00

**Table 5:** The prediction results of two RVTR models that were tested on different sized networks with 10%, 20% and 30% nodes removed, respectively.

Removed	Model	Network	1000	2000	3000
10%	RVTR-1K	Precision	0.68 ± 0.03	0.62 ± 0.02	0.55 ± 0.02
		AUC	0.99 ± 0.01	0.99 ± 0.01	0.97 ± 0.03
	RVTR-3K	Precision	**0.73 ± 0.04**	**0.70 ± 0.01**	**0.66 ± 0.02**
		AUC	0.98 ± 0.03	0.99 ± 0.01	0.91 ± 0.16
20%	RVTR-1K	Precision	0.89 ± 0.02	0.83 ± 0.02	0.80 ± 0.01
		AUC	0.98 ± 0.02	0.99 ± 0.01	0.98 ± 0.01
	RVTR-3K	Precision	**0.91 ± 0.02**	**0.88 ± 0.01**	**0.87 ± 0.01**
		AUC	0.98 ± 0.02	0.99 ± 0.01	0.96 ± 0.06
30%	RVTR-1K	Precision	0.98 ± 0.01	0.97 ± 0.01	0.97 ± 0.01
		AUC	0.98 ± 0.02	0.99 ± 0.00	0.99 ± 0.00
	RVTR-3K	Precision	0.98 ± 0.01	**0.98 ± 0.00**	**0.98 ± 0.00**
		AUC	0.99 ± 0.01	0.98 ± 0.02	0.99 ± 0.00

**Table 6:** The results in the Australia dataset (training and testing both on the Australia dataset). The tests were performed on 10%, 20% and 30% nodes removed, respectively.

Removed	Metric	RVTR	GCN	SDNE	AE	PCA	DIRECT
10%	Precision	**0.40 ± 0.07**	0.24 ± 0.04	0.25 ± 0.05	0.23 ± 0.05	0.29 ± 0.03	0.26 ± 0.04
	AUC	0.99 ± 0.01	0.85 ± 0.28	0.50 ± 0.00	0.50 ± 0.00	0.45 ± 0.18	0.44 ± 0.22
20%	Precision	**0.59 ± 0.03**	0.46 ± 0.05	0.47 ± 0.04	0.46 ± 0.05	0.53 ± 0.03	0.49 ± 0.02
	AUC	0.96 ± 0.03	0.74 ± 0.37	0.50 ± 0.00	0.50 ± 0.00	0.41 ± 0.16	0.33 ± 0.09
30%	Precision	**0.75 ± 0.04**	0.69 ± 0.03	0.70 ± 0.02	0.68 ± 0.04	0.72 ± 0.01	0.71 ± 0.10
	AUC	0.95 ± 0.05	0.63 ± 0.43	0.50 ± 0.00	0.50 ± 0.00	0.48 ± 0.22	0.34 ± 0.10

**Table 7:** The test results in different datasets (model trained on the Australia dataset and tested on other datasets).

Dataset	RVTR	GCN	SDNE	AE	PCA	Direct
NZ	**0.7904**	0.6651	**0.8064**	0.6720	0.6720	**0.8009**
CA	**0.6654**	0.5000	0.5627	0.5627	0.6103	0.5760
AB	**0.5399**	0.3599	0.4348	0.4348	0.4855	0.4469
NY	**0.9363**	0.8916	0.9186	0.8916	0.9143	0.9186

**Table 8:** The comparison of test results on two networks generated by different R value.

Removed	Metric	R1.7	R3	R4.8
10%	Precision	0.32 ± 0.06	**0.73 ± 0.04**	0.72 ± 0.05
	AUC	0.73 ± 0.17	0.98 ± 0.03	0.80 ± 0.29
20%	Precision	0.54 ± 0.06	0.91 ± 0.02	**0.98 ± 0.01**
	AUC	0.81 ± 0.14	0.98 ± 0.02	0.67 ± 0.24
30%	Precision	0.74 ± 0.05	0.98 ± 0.01	**0.99 ± 0.01**
	AUC	0.80 ± 0.19	0.99 ± 0.01	0.66 ± 0.24

**Figure A1:**
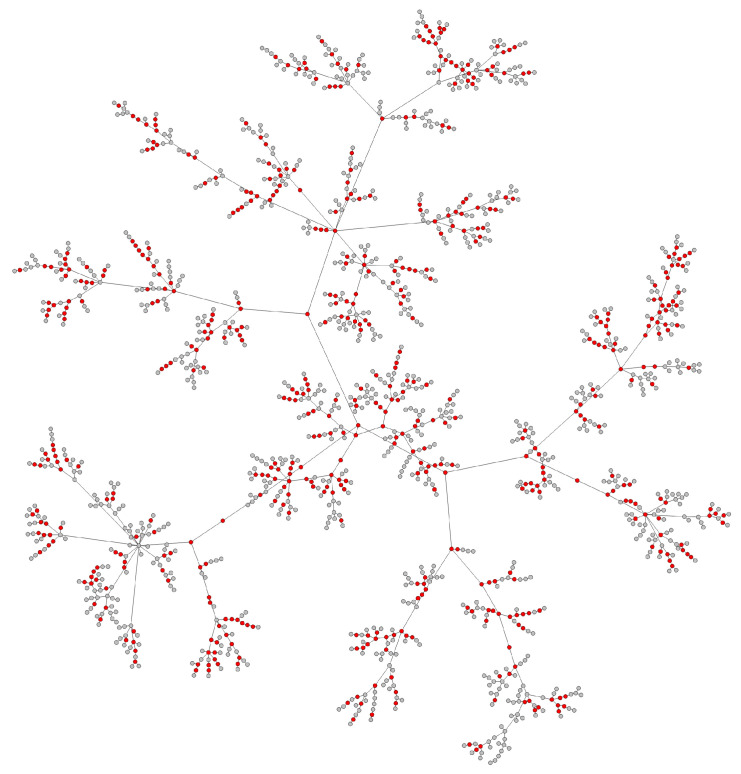
The inferred Canada transmission network.

**Figure A2:**
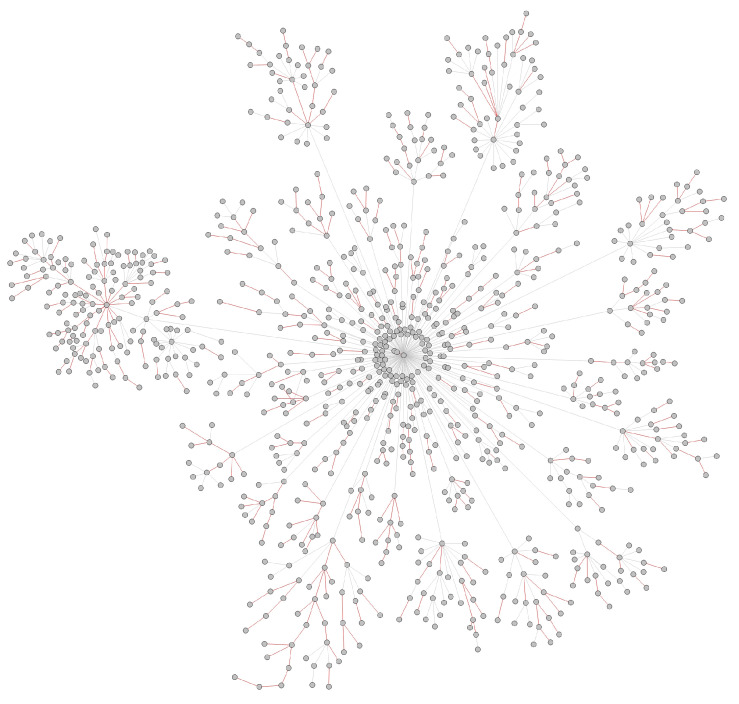
Abnormal edges in Canada transmission network.

**Figure A3:**
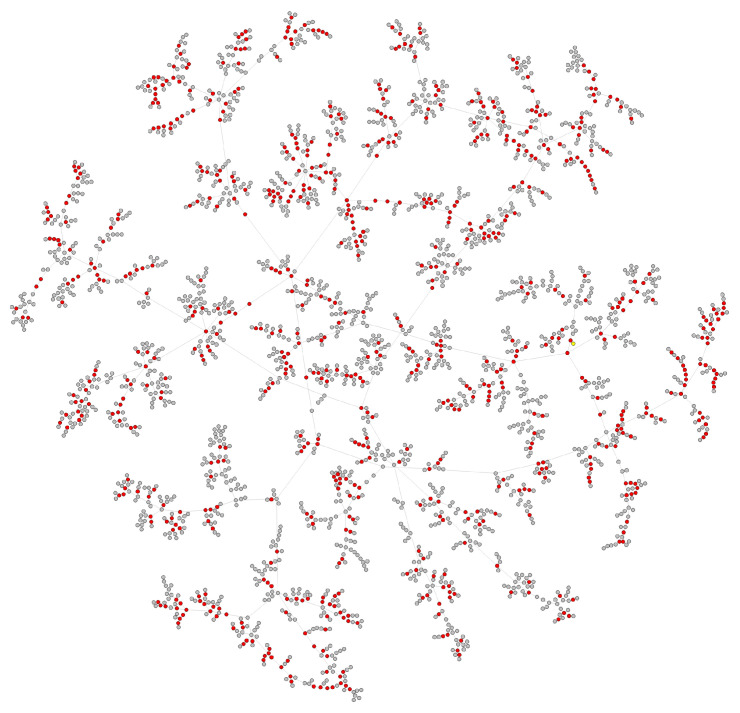
The inferred Alberta transmission network.

**Figure A4:**
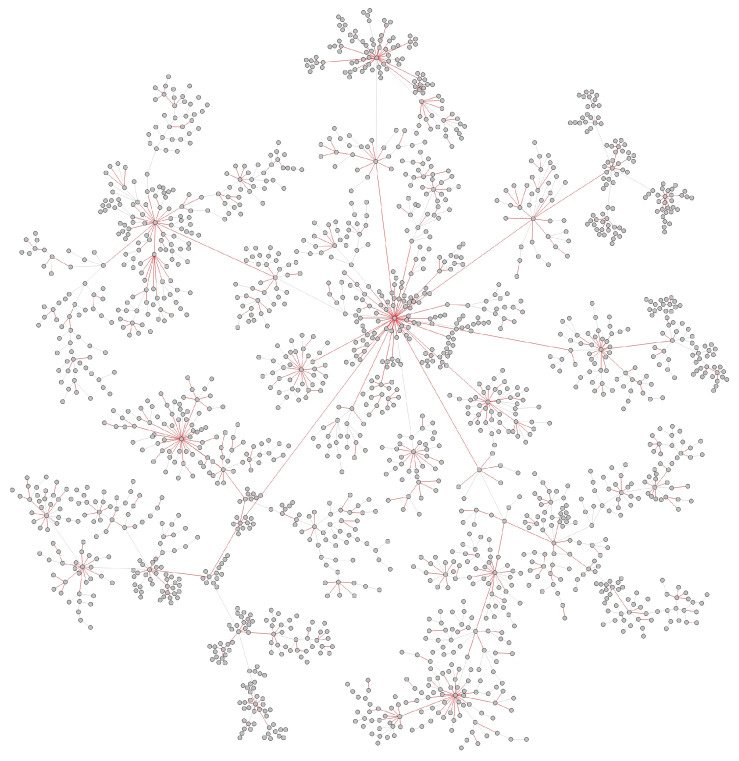
Abnormal edges in Alberta transmission network.

**Figure A5:**
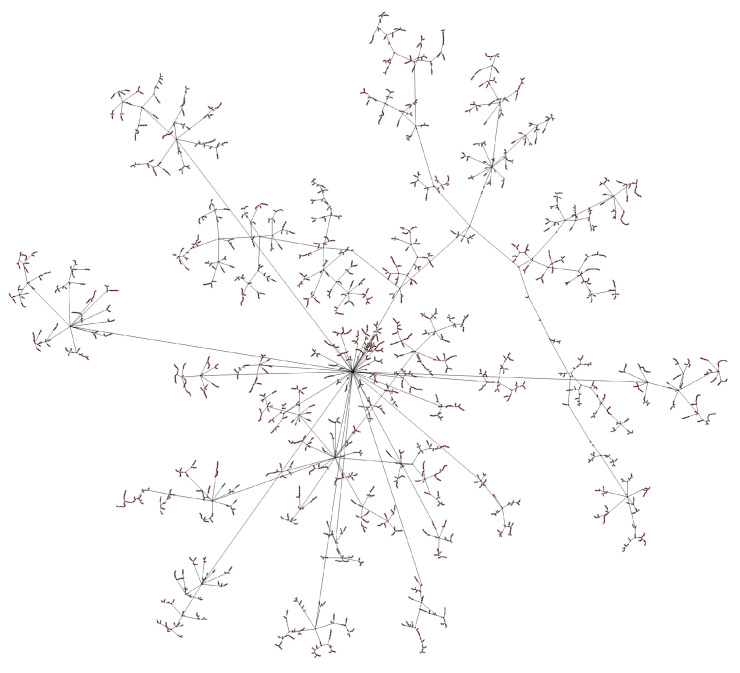
The inferred New York State transmission network.

**Figure A6:**
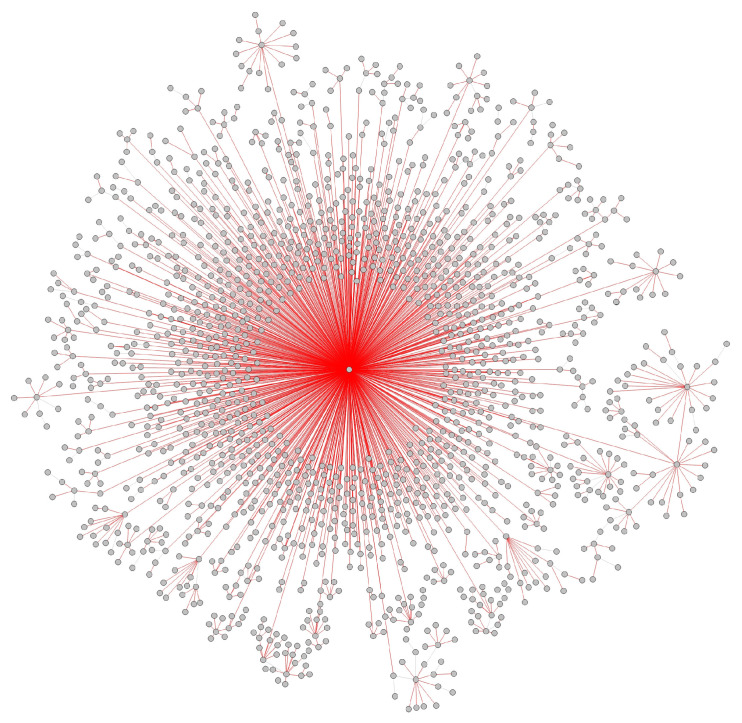
Abnormal edges in New York State transmission network.

**Figure A7:**
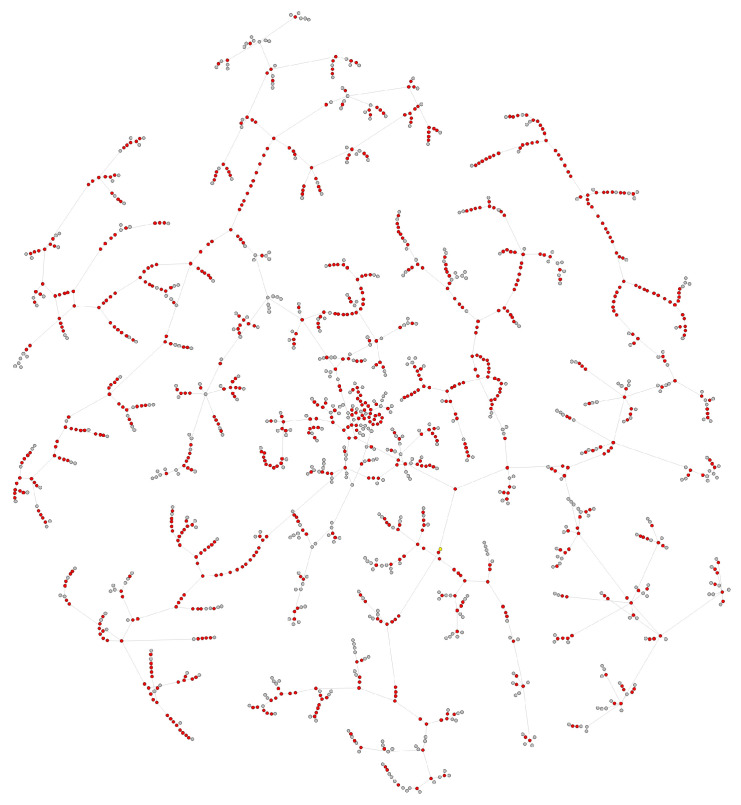
The inferred New Zealand transmission network.

**Figure A8:**
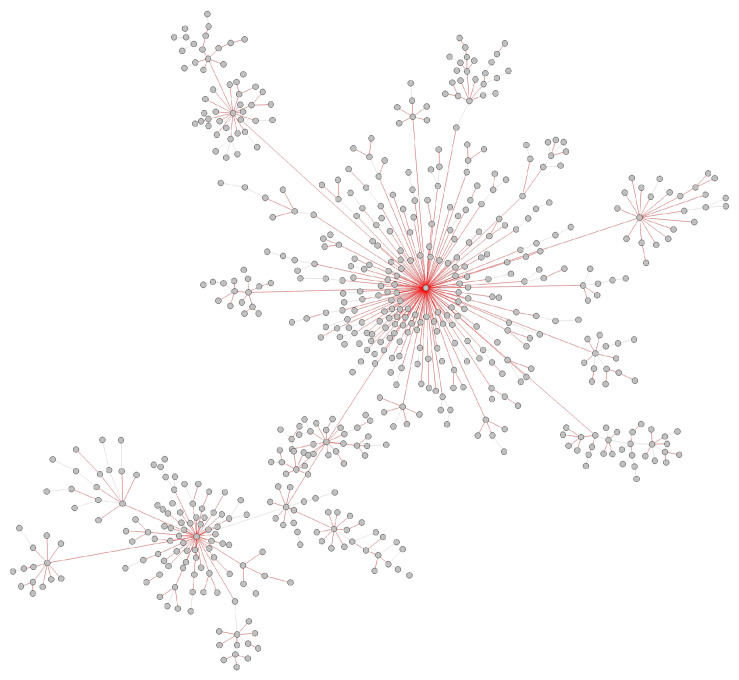
Abnormal edges in New Zealand transmission network.

## Data Availability

Publicly available datasets were analyzed in this study. This data can be found here: https://github.com/lzylyn/COVID-19-Transmission-Network.

## Abbreviations

The following abbreviations are used in this manuscript:

COVID-19	Coronavirus disease 2019
RVTR	RNA virus transmission network representation model
GCN	Graph convolutional neural network
SDNE	Structural deep network embedding
PCA	Principal component analysis
AE	Autoencoder

## Appendix A

### Appendix A.1. Comparison Algorithm Description

We used 2 network representation methods and 3 feature extraction methods as comparison algorithms.

- GCN uses a low pass filter to generate node embedding; the information of each node is transferred to the neighbor nodes in the graph, and the graph can transfer information layer by layer through a convolution operation.
- SDNE uses a deep neural network to model the nonlinearity between node representations. The whole model can be divided into two parts: the modeling module of level 1 similarity supervised by the Laplace matrix and the modeling of the level 2 similarity relation by an unsupervised deep autoencoder.
- PCA is one of the most widely used data dimension reduction algorithms. The main idea is to map the possibly correlated N-dimensional feature data to the linearly unrelated K-dimension through orthogonal change, reconstruct the K-dimensional features on the basis of the original N-dimensional features, and then achieve efficient and accurate representation of the original data. The transformed K-dimensional features are called principal components.
- AE consists of an encoder and a decoder. The encoder can compress the input attributes into potential spatial representations, and the decoder can reconstruct the data input from potential spatial representations. Autoencoders are often used for dimensionality reduction or feature learning.
- DIRECT is to use the gene sequence directly to calculate node similarity. After the gene sequence of each node was encoded by a one-hot encoder, it was directly used to calculate the similarity score for each pair node without dimension reduction.

### Appendix A.2. Reproducibility

For the proposed model, the training epoch was set as 3000. We started with a learning rate of 0.01 for training, which was divided by a factor of 2 every 200 epochs. The output dimension of the RVTR was set as 256, and the dimension of the hidden layer was 512. Regularization parameter β was 0.001, attention amount $n_{h}$ was 4, the seed of random was 1, the length of random walk in the network $L_{w}$ was 10, and the restart probability of random walk $p_{r}$ was 0.2.

### Appendix A.3. Real Data Description

- **Data resource:** Both FASTA sequence data and the corresponding epidemiological data of Canadian, New Zealand, New York State, and Australia were downloaded from GISAID (https://www.gisaid.org/ Accessed on 1 September 2020.) [26]. We confirmed that the selected data had complete sequences with high sequence coverage. The length of complete sequence comprised of genoms is greater than 29,000, the proportion of undefined bases for high coverage sequences was less than 1%, while it was greater than 5% for low coverage sequences.
- **Data description:** The data collection of the Canadian dataset is from January 2020 to July 2020, and there was a high sampling rate with over 4 million tests being performed (with a weekly average of over 55,000 tests at 0.8% positivity), thus ensuring that the dataset is concise. The sampling time of the Australia dataset was from January 2020 to April 2020. The sampling date of New Zealand is from March 2020 to January 2021, and the sampling time span was the longest in these datasets. However, half of the sampled infections were detected in March and April of 2020, and there were quite few infections sampled in May, June and July of 2020. This spread is unique when compared with other transmission datasets. During the study period (February 2020 to September 2020), New York State was considered a COVID-19 epicenter with the second-highest case numbers of COVID-19 in the USA in March 2020; however, it had a lower sampling rate in comparison to its rate of disease incidence [10]. The sampling time of the Alberta dataset was from March 2020 to May 2020.

### Appendix A.4. Real Data Process

- **Sequence generation for patient “0”**
  As the transmission network inferred by TransPhylo was based on the assumption that all infections have a common ancestor, we needed to simulate the gene sequence of patient “0” for each inferred transmission network before we trained and tested our model on them. If we removed patient “0” from the network, the transmission network collapsed into several disconnected small networks. The basic idea to simulate the sequence for patients “0” was also based on the mutation of SARS-CoV-2 virus; all of the individuals in a local module in a network share a common quasi-species signature of the gene sequence. We sought the nearest neighbor node of patient “0” in the inferred transmission network, then copied its sequence and changed some genes randomly to reflect the difference between patient “0” and its child nodes.
- **Sequence alignment for different test data**
  The length of the sequence in the FASTA data is slightly different from dataset to dataset, and the different sequence data need to be adjusted for alignment before being used in the experiments. As our trained model is based on the Australia dataset, we used ‘-’ to fill the sequence whose length is shorter than that of Aussies. For the sequences in a common dataset, the number and location of newly added genes ‘-’ were the same, so this operation had no effect on the test dataset.
- **Transmission networks of real data**
  In this section, we show the transmission networks of different regions. Transmission networks were visualized using Gephi 0.9.2 which is a network analysis software [30]. Gephi’s built-in clustering algorithms Force Atlas 2 [31] is used to identify population clusters in the transmission network.
  In these figures, the red nodes represent inferred undetected spreaders in a transmission network. The red edges are abnormal edges with undetected nodes in them and are also used as test labels. Figure 7, Figure A1, Figure A3, Figure A5 and Figure A7 show the inferred transmission networks of Australia, Canada, Alberta, New York State and New Zealand respectively. Figure 8, Figure A2, Figure A4, Figure A6 and Figure A8 shows the observed transmission networks of Australia, Canada, Alberta, New York State and New Zealand respectively, which were also used as our test network. From these figures, we can see that the spread in different from region to region, and the distribution of the number of abnormal edges is different.
